# Autoimmune thyroid disease and ovarian hypofunction: a review of literature

**DOI:** 10.1186/s13048-024-01451-y

**Published:** 2024-06-14

**Authors:** Ru Wang, Youyuan Lv, Tao Dou, Qian Yang, Chunxiao Yu, Qingbo Guan

**Affiliations:** 1 Key Laboratory of Endocrine Glucose & Lipids Metabolism and Brain Aging,Ministry of Education, Department of Endocrinology, Shandong Provincial Hospital of Affiliated to Shandong First Medical University, Jinan, 250021 Shandong China; 2 Shandong Key Laboratory of Endocrinology and Lipid Metabolism, Jinan, 250021 Shandong China; 3 Shandong Institute of Endocrine and Metabolic Diseases, Jinan, 250021 Shandong China; 4 Shandong Engineering Laboratory of Prevention and Control for Endocrine and Metabolic Diseases, Jinan, 250021 Shandong China; 5 Internal Medicine Department of the Second Affiliated Hospital of Shandong University, Jinan, 250021 Shandong China

**Keywords:** Autoimmune thyroid disease, Hyperthyroidism, Hypothyroidism, Ovarian, Premature ovarian failure

## Abstract

Thyroid hormones(THs) are essential for the proper functioning of the ovaries, and multiple studies have shown that thyroid abnormalities, especially during adolescence and reproductive age, can lead to lifelong ovarian dysfunction. Autoimmune thyroid disease (AITD), one of the most common organ specific autoimmune diseases, is mainly mediated by cellular autoimmune reactions, and has strong inflammatory infiltration and immune active cells, including chemokines and cytokines, which are important components of ovarian aging. This suggests that autoimmune and inflammatory molecular processes may play a role in the emergence of ovarian dysfunction. The purpose of this review is to summarize recent in vivo and in vitro evidence of a complex relationship between AITD and ovarian dysfunction. AITD is closely related to the decline of ovarian function from the perspective of antibody, cytokine, oxidative stress, and genetic factors. Finally, some of the currently known treatments for AITD and hypo ovarian disease are summarized.

## Introduction

1% of women may experience reproductive senescence before age 40 for a variety of reasons, a condition called premature ovarian failure (POF) and is defined secondary to low serum estrogen levels and high gonadotropin levels during menopause [1]. In addition to traditional diagnostic markers, anti-Mullerian hormone (AMH) is an alternative diagnostic marker for determining ovarian reserve function in women [2]. The consequences of POF include decreased fertility and an increased risk of osteoporosis, cardiovascular disease, dementia, decline in cognition, and Parkinson’s syndrome [3–7]. At present, hormone replacement therapy (HRT) is used in clinical practice to alleviate low estrogen symptoms in patients with POF, and assisted reproductive technology is used to solve fertility problems [8–10]. However, there is no effective treatment measure to improve ovarian function. Patients with POF usually have two or more autoimmune diseases. Even recurrent pregnancy loss in human beings is definitely associated with polyautoimmunity [11]. According to reports, the most common form of POF-associated autoimmune disease is thyroid disease, with approximately 12–33% of POF patients presenting with varying degrees of Autoimmune thyroid disease (AITD) [12–15]. 

AITD is one of the most common organ-specific autoimmune diseases with lymphocytes dysfunction caused by infection, trauma, and other stress factors based on heredity [16]. Hashimoto’s thyroiditis (HT) is the most frequent AITD, with a prevalence of about 18% among female adults [17]. Autoimmune thyroiditis can also manifest as hyperthyroidism (Graves’ disease, GD). It is characterized by abnormal increase or decrease of thyroid hormones and positive thyroid autoantibodies. There are also some patients with normal thyroid hormone levels, only showing positive antibodies. AITD leads to decreased fertility and an increased risk of pregnancy loss [18]. Increased risk of decreased ovarian reserve function in AITD-afflicted women of reproductive age [19–21]. Autoimmune thyroid disease may be an important risk factor for ovarian dysfunction. In the present review, we aim to provide an update review on the role of autoimmune thyroid disease in ovarian dysfunction.

## Thyroid hormones (THs) is essential for normal ovarian function

Thyroid hormone receptors are expressed in human oocytes, thyroid hormone may affect ovarian function by directly acting on oocytes [22]. In humans, the expression of Thyroid hormone receptors is found to increase with the growth of follicles [22, 23]. It was confirmed through in vitro research that THs stimulate the growth of preantral follicles in rats, supporting the idea that THs may directly affect the ovaries [24]. In addition, thyroid hormone can affect ovarian function by influencing the release of gonadotropin-releasing hormone and Follicle-stimulating hormone (FSH) from the hypothalamus-pituitary-gonadal axis through negative feedback. It can also indirectly affect ovarian function by acting on insulin-like growth factor, prolactin and estrogen [25]. 

The steroid hormones released by granulosa cells are crucial for the normal development of follicles [26]. The interaction of T3 with the gonadotrophin hormone inhibiting excessive production of androgens by follicular membrane cells while simultaneously stimulating granulosa cells to secrete estrogen [27]. Estradiol, testosterone, and dihydrotestosterone are transported through the bloodstream via sex hormone-binding globulin (SHBG) [28]. THs can also affect the bioavailability of sex steroids by regulating SHBG [29]. Studies showed that the serum T3 and T4 values were positively correlated with the follicular triiodothyronine (T3) and thyroxine (T4) values [30, 31]. Therefore, changes in serum THs levels, such as hypothyroidism and hyperthyroidism, may lead to ovarian hypofunction. T3 can also influence follicle development by stimulating the proliferation of granulosa cells and inhibit apoptosis by activating the PI3K/Akt pathway [32]. This signaling pathway is involved in regulating the dormancy and activation of primordial follicles [33, 34]. T3 has been shown to shield ovarian granulosa cells against chemotherapy [35]. 

## Thyroid autoantibodies affect ovarian function

Thyroid autoimmune disease is an organ-specific autoimmune disease caused by humoral and cellular immunity. Many studies have shown that the autoimmune course of the disease begins with the specific activation of helper T cells (CD4) against thyroid antigens, stimulating autoreactive B cells to accumulate in thyroid tissue and secrete anti-thyroid antibodies [36–39]. Thyroid autoantibodies may can damage thyroid tissue by relying on cell-mediated cytotoxicity and altering the function of target cells, but this claim is controversial and has not been directly proven [40–42]. Reportedly, 40% of women with POF are positive for at least one organ-specific autoantibody, the most common being antithyroid antibodies (20%) [43]. In a recent meta-analysis, the authors confirmed that the positive rate of anti-thyroid peroxidase antibodies (TPO-Ab) was higher in POF patients but not in anti-thyroglobulin antibodies (TgAb) [44]. The presence of TPO-Ab is associated with decreased ovarian function [45], which may have the following mechanisms: [46] (i) Thyroid autoantibodies are an epiphenomenon of generalized immune dysfunction [47]. TPO-Ab levels only represent differences in autoimmune levels and that other factors in the autoimmune process cause a decline in ovarian function. (ii) The decline in ovarian function is secondary to changes in THs. TPO-Ab can cause chronic lymphocytic thyroiditis, causing THs to fail to regulate ovarian function normally [48]. (iii) This association is affected by age, because the positive rate of TPO-Ab increases with age, and women’s ovarian function gradually declines with age, but this assumption is controversial [19, 49]. The presence of anti-thyroid antibodies (TgAb and TPO-Ab) in follicular fluid of AITD women was confirmed in a study involving 5000 people, and serum antibody levels were positively correlated with follicular fluid autoantibodies [50]. The concentration of antithyroid antibodies in follicular fluid is about half that of serum antithyroid antibodies and ultimately leads to ovarian dysfunction [51]. This mechanism may be related to the fact that antithyroid peroxidase and antithyroid globulin antibodies can cross the blood follicle barrier during follicular maturation and cause cytotoxic environmental damage to mature oocytes [50]. 

In addition, the zona pellucida(ZP) and thyroid tissue seem to share similar antigens. Therefore, some studies infer that the zona pellucida is the target of the thyroid antibody [52, 53]. In mouse tissue, anti-zona pellucida antibodies cross-react only with the thyroid gland [53]. Bidirectional communication between oocytes and granulosa cells is achieved through microvilli and interstitial junctions in the ZP. The abnormal structure and function of ZP interfere with this communication process, resulting in ovarian dysfunction. Therefore, thyroid antibodies may act on ZP in the ovary and induce autoimmune ovarian disease and POF through anti-zona pellucida antibodies.

## AITD reduces ovarian function by affecting cytokines

Cytokines play an important role in the immunopathology of AITD. As major cytokines, T lymphocytes are divided into the Th1 group and Th2 group. Th1 cells induce disease and accelerate disease progression. Th2 cells can prevent and alleviate disease. In AITD patients, Th1 cytokine hyperactivity in the early stages is predominant [36]. In addition, Th17 cells also play a highly relevant role in the pathogenesis of chronic inflammation and tissue damage observed in AITD, such as IL-17 and IL-22 [54–56]. Karanikas et al. demonstrated for the first time that thyroid peroxidase antibody titers were associated with Th1 cytokine production in patients with AITD and that groups with high antibody titers produced more Th1 factors such as Tumor Necrosis Factor alpha (TNF-α) and Interferon-gamma (IFN-γ) than groups with low antibody titers and controls [57]. TNFα may accelerate atresia by inhibiting the action of gonadotropins and blood supply to the ovarian follicle [58, 59]. Follicular atresia is encouraged by IFN-γ, which also causes the up-regulation of apoptosis-promoting Fas molecules [60]. As a result, higher antibody titers in AITD can impair ovarian function by increasing the production of pro-inflammatory cytokines including TNF-α and IFN-γ.

Regulatory T cells (Tregs) play an important role in regulating the immune system’s immune response to autoantigens and can suppress the immune response by secreting immunosuppressive cytokines [61]. But the percentage of Treg cells (CD4 + CD25 + Foxp3+) was significantly decreased in the spleen of autoimmune ovarian disease mice. Upregulating the number of Treg cells in the spleen can improve inflammatory response and restore damaged ovarian function [62]. Changes in T-cell subsets and T-cell-mediated immune impairment in patients with early ovarian insufficiency are indicated by decrease of CD4 + T cells, increment of CD8 + T cells, and reduction of CD4+/CD8 + ratio [63]. Cytokines expressed and secreted by T cells act on B cells to produce antibodies that destroy the follicle, leading to a decrease in the number of follicles. These suggest that cellular immunity also plays a key role in the destructive autoimmune response of the ovary.

## AITD induces oxidative stress and reduces ovulation rate

Positive thyroid autoantibodies represent the activation of the immune system, activate complement or antibody-dependent cytotoxicity. This pathological process triggers excessive production of reactive oxygen species (ROS) which leads to increased oxidative stress in target organs [64, 65]. ROS is produced during normal ovarian metabolism and is an essential substance involved in ovarian physiological activities in a balanced state. But once the balance between oxidative stress and oxidative defense is disrupted, the side effects of ROS will overwhelm its physiological function, ultimately affecting follicular development and atresia, cell apoptosis as well as other cell activities [66]. Previous reports have shown that superoxide dismutase 1 (SOD1) mRNA and protein content were significantly increased in the ovaries of hypothyroid mice, and the cytoplasmic antioxidant gene catalase mRNA and protein content were significantly decreased [67, 68]. These changes indicate that the ovarian cells of hypothyroid animals have decreased antioxidant defense capabilities and increased oxidative stress levels. The significant reduction in the expression of mitochondrial antioxidant enzyme peroxidase protein 3 (Prdx3) and cytoplasmic antioxidant enzyme glutathione peroxidase 3 (Gpx3) can also support the above view. In the ovaries of hypothyroid mice, strong staining of oxidative stress marker 4-hydroxynonenal (4-HNE) in atretic follicular membrane cells, interstitial cells and corpus luteum were observed, suggesting the existence of oxidative stress [69, 70]. These results suggest that the reduced ovulation rate in AITD is associated with disruption of the ovarian antioxidant defense system.

Thyroid hormone administration can induce activation of Nrf2 in the rat liver, and this activation can be inhibited by the ROS scavenger N-acetylcysteine [71]. Nuclear factor erythroid 2- related factor 2 (Nrf2) is a member of the cap-n-collar subdivision of the basic domain-leucine zipper-type enzyme family responsible for regulating transcriptional frequency. Its function is to induce the production of cell defense proteins [72]. Nrf2 is highly expressed in ovarian tissues of reproductive age (8–12 weeks old) mice, mainly located in the granulosa cells, secondary follicles and antral follicles of oocytes, and some research results indicate that Nrf2 protein signaling is necessary for the antioxidant process during ovarian aging [73]. In the same line, a recent study showed that in the lymphatic tissues of thyroid toxic mice treated with high-dose T4, ROS levels increased, and then Nrf2 was activated, and the transcription of genes encoding antioxidant enzymes was upregulated [74]. The activation of Nrf2 by THs has been further confirmed in studies on the heart [75] and liver [76]. Although few studies have been conducted on the ovaries, the above analysis suggests that thyroid autoimmune diseases may affect Nrf2, leading to abnormal ovarian function [77]. 

## Genetic correlation between AITD and ovarian dysfunction

Genetic factors are important in the pathogenesis of autoimmune thyroiditis. Using the candidate gene method, we conclude that the HLA-DR3, cytotoxic T-lymphocyte-associated protein 4 and TSHR genes are the major susceptibility genes for GD and HT [78]. Among them, the HLA-DR3 haplotype is associated with increased risk of POF [79]. Methylenetetrahydrofolate reductase (MTHFR) catalyzes the conversion of 5,10-methylenetetrahydrofolate to 5-methyltetrahydrofolate, providing methyl groups in some methylation reactions [80]. DNA hypermethylation produced by MTHFR may cause AITD, leading to pathological changes in thyroid function [81]. The two most common genetic polymorphisms of the MTHFR gene are g.677 C > T and g.1298 A > C variants. But study has shown that MTHFR C667T/A1298C genotypes are not associated with POF development [82]. There is also different evidence that during follicular development, granulosa cells respond to FSH for proliferation, and the ovarian response to FSH stimulation and AMH levels in patients may be related to MTHFR polymorphism [83, 84]. These findings provide evidence for the regulation of follicular development and ovarian reserve by MTHFR A1298C polymorphism. One of the mechanisms by which genetic and environmental risk factors jointly promote AITD is through epigenetic control of gene expression. There is still very little research in this field. However, there has been wide confirmation of the role of X chromosome inactivation [85]. However, according to several studies, X chromosome inactivation may not be associated with idiopathic POF [86, 87]. 

FMR1 gene mutations and autoimmune induction account for a majority of POF cases [88, 89]. Phenotypic expression of aberrant triple CGG amplification of the FMR1 gene includes abnormal increases in FSH levels, premature menopause, and premature ovarian failure [88]. To date, the underlying mechanism of FMR1 CGG repeat-relevant ovarian function modulation has not been well investigated. One hypothesis is that during normal follicular development, FMRP is mainly expressed in granulosa cells, which is crucial for the maturation and growth of oocytes. Expansion of FMR1 CGG may lead to changes in transcription levels, reducing FMRP levels, thereby affecting the expression of steroidogenic enzymes and hormone receptors, ultimately affecting the occurrence of POF [90, 91]. The number of triple repeats in the FMR1 gene in autoimmune patients is statistically low, indicating that the risk of ovarian aging may not be related to mutations in this gene. This means that abnormal autoimmune function itself represents a risk of premature ovarian senescence [92]. It is also believed that cross-reactive epitopes with other endocrine organs most commonly involved in the thyroid gland may be responsible for the aberrant immune response of POF [93]. Previous observations have shown an increased risk of premature ovarian failure with an increase in the number of triple CGG amplifications [92, 94]. The above study indicates that abnormal autoimmune function and excessive CGG triple duplication of the FMR1 gene can independently induce premature ovarian failure [94]. This means that AITD women with CGG amplification ≥ 30 for the FMR1 gene clinically are at risk for premature ovarian senescence and therefore a prospective follow-up should be performed to find early clinical evidence to confirm this diagnosis [92]. 

## Current treatments

Up to now, HRT has been mainly used clinically to maintain a series of clinical symptoms caused by ovarian hypofunction [95]. There are also studies advocating gene and immunotherapy [18, 96–98]. There is increasing evidence that Stem Cell Therapy is a potential treatment for POF disorders. Heme oxygenase-1 in umbilical cord mesenchymal stem cells activates autophagy regulated by the JNK/Bcl-2 signaling pathway, upregulates the CD8 + CD28-T cell cycle, and restores ovarian function in mice with POF [99–101]. Another study has shown that transplantation of human placenta-derived mesenchymal stem cells can restore ovarian function impairment induced by ZP3 immunity in mice [102]. The process of restoration of function is related to the proportion of Th17/Treg and Th17/Tc17 cells and the balance of the PI3K/Akt signaling pathway [103]. Stem cells may also provide therapeutic effects through paracrine signaling, and exosomes show similar regeneration promoting characteristics with stem cells. Direct treatment with exosomes can avoid many adverse reactions of stem cell transplantation [104]. The miR-369-3p carried by exosomes from human amniotic fluid stem cells can inhibit the expression of proteins such as YY1-associated factor 2, programmed cell death protein 5, and p53, reduce apoptosis of mouse ovarian granulosa cells, and thus exert therapeutic effects on premature ovarian failure [105]. 

At present, treatment studies in women with thyroid antibody positive are levothyroxine and intravenous immunoglobulin. Intravenous immunoglobulin may modulate the transition from Th1 to Th2 cell response [106]. Adjusting the ratio of th1/th2 cell subgroups and correcting the imbalanced cytokines may become a new approach for immune prevention and treatment of AITD [107]. In a study that directly compared levothyroxine versus intravenous immunoglobulin therapy, women treated with levothyroxine had a higher live birth rate [108]. Similarly, the American Thyroid Association guidelines recommend that women with normal thyroid function who are antibody-positive and have recurrent miscarriages should not use intravenous immunoglobulin [109]. In addition, based on the importance of MTHFR polymorphism, future studies should include larger sample sizes to establish associations, thus allowing the incorporation of MTHFR polymorphisms into screening of susceptible individuals. Once the mechanism and statistical relationship are established, clinicians introduce preventive guidelines for susceptible patients, and artificial supplementation of 5-methyltetrahydrofolate can reduce the risk of AITD by replacing the reduced MTHFR function [110]. 

Studies have shown that after thyroxine treatment, granulosa cells can increase gonadotropin-induced estradiol and progesterone production and promote ovarian follicle development in immature hypothyroidism rats [23, 111, 112]. The development of mature ovarian follicles is greatly dependent on healthy thecal angiogenesis. Recent experimental evidence showed that thyroxine may promote ovarian follicular angiogenesis by up-regulating mRNA expression of major angiogenic factors [112]. 

## Conclusion and future directions

**Fig. 1:**
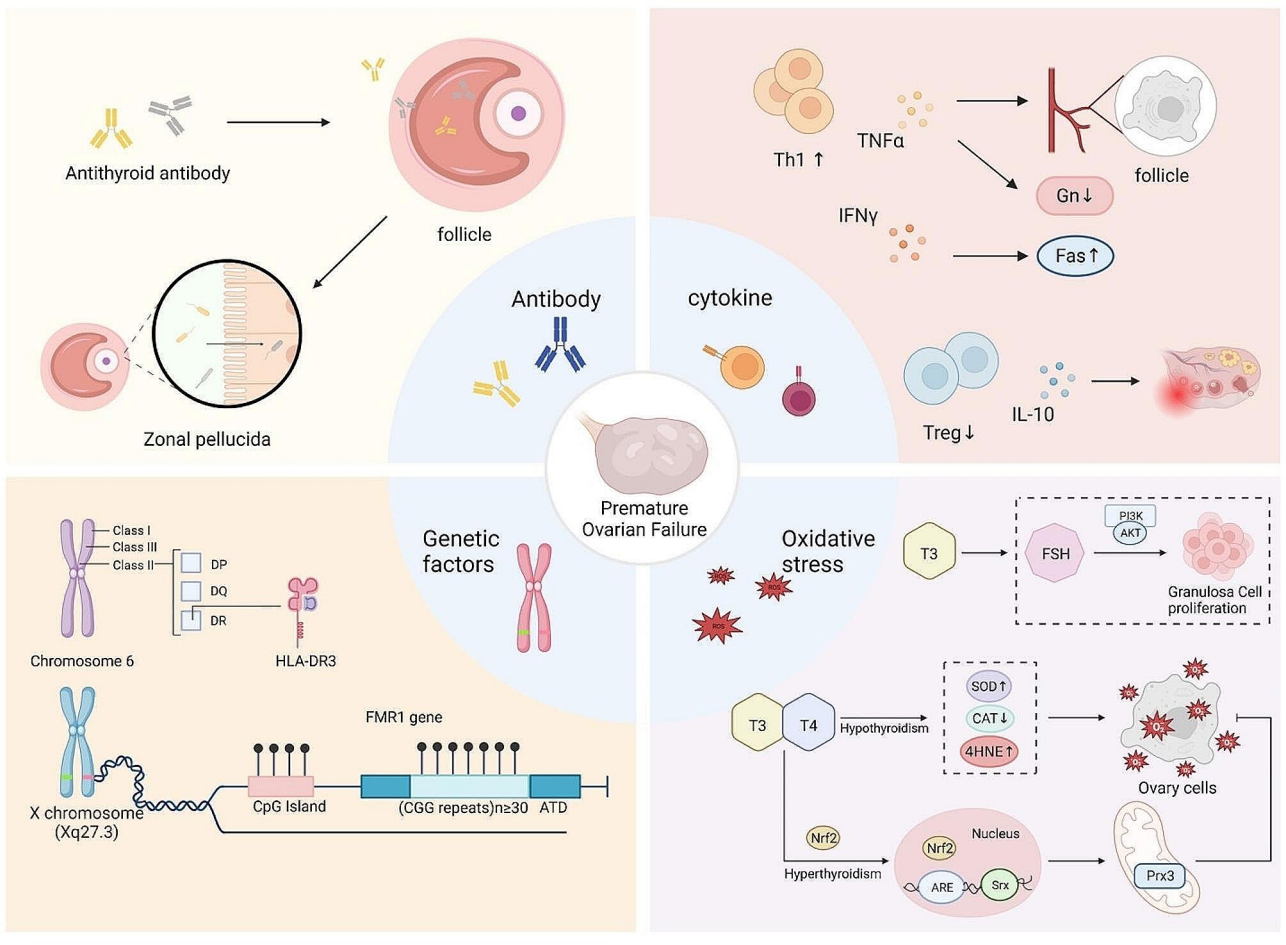
Schematic diagram of the possible mechanism of autoimmune Thyroid disease affecting normal ovarian function through antibodies, cytokines, oxidative stress and genes. **a.** Thyroid antibodies cross the blood follicle barrier and affect the microenvironment of follicles and target the zona pellucida, thereby affecting ovarian function. **b.** Cytokines secreted by Th1 accelerate follicular atresia by inhibiting the effect of Gonadotropin, the blood supply of follicles, and inducing the up regulation of apoptosis-promoting Fas molecule. The weakened effect of T regulatory lymphocytes on inhibiting inflammation affects ovarian function. **c.** During oxidative stress, Nrf2 penetrates the nucleus, and then Nrf2 attaches to the antioxidant response element, which activates the expression of the antioxidant genes Srx and Prx 3, resulting in enhanced antioxidant defense. **d.** The HLA-DR3 haplotype is associated with increased risk of premature ovarian failure. Ovarian dysfunction caused by FMR1 gene CGG amplification ≥ 30 in women with autoimmune Thyroid disease. T4: L-thyroxine. T3: L-triiodothyronine. IFNγ: Interferon γ. TNFα: Tumor Necrosis Factor-α. Gns: Gonadotropins. Fas: factor associated suicide. IL-10: Interleukin-10. ARE: antioxidant response elemen

In summary, recent findings highlight the close association between AITD and ovarian dysfunction, supporting that AITD may be directly or indirectly related to ovarian maturation and normal function through antibodies, cytokines, oxidative stress, and genes (Fig. 1). Given the complexity of its function, THs may affect ovarian function through completely different mechanisms. Nevertheless, most of the reviewed studies were conducted in animal models, and this limitation is also worthy of attention. It is becoming increasingly clear that thyroid antibodies and THs affect various metabolic pathways in the ovary through general activation of immune responses and specific regulation of various pathways. Currently, the relationship between AITD and decreased ovarian function is not yet fully understood. Therefore, an in-depth understanding of the potential damage of AITD to follicles in various aspects is of great importance for the prevention and treatment of ovarian diseases accompanied by thyroid dysfunction.

## Data Availability

Not applicable.

## Abbreviations

4-HNE: 4-hydroxynonenal
AITD: Autoimmune thyroid disease
ARE: Antioxidant response element
FSH: Follicle-stimulating hormone
GD: Graves’ disease,
Gpx3: Glutathione peroxidase 3
HRT: Hormone replacement therapy
HT: Hashimoto’s thyroiditis
IFN-γ: Interferon-gamma
MTHFR: Methylenetetrahydrofolate reductase
Nrf2: Nuclear factor erythroid 2- related factor 2
POF: Premature ovarian failure
Prdx3: Peroxidase protein 3
ROS: Reactive oxygen species
SOD1: Superoxide dismutase 1
T3: Triiodothyronine
T4: Thyroxine
TgAb: Anti-thyroglobulin antibodies
THs: Thyroid hormones
TNF-α: Tumor Necrosis Factor alpha
TPO-Ab: Anti-thyroid peroxidase antibodies
Tregs: Regulatory T cells
ZP: Zona pellucida
